# Ten simple rules for providing effective bioinformatics research support

**DOI:** 10.1371/journal.pcbi.1007531

**Published:** 2020-03-26

**Authors:** Judit Kumuthini, Michael Chimenti, Sven Nahnsen, Alexander Peltzer, Rebone Meraba, Ross McFadyen, Gordon Wells, Deanne Taylor, Mark Maienschein-Cline, Jian-Liang Li, Jyothi Thimmapuram, Radha Murthy-Karuturi, Lyndon Zass

**Affiliations:** 1 H3ABioNet, Centre for Proteomic and Genomic Research, Cape Town, South Africa; 2 Iowa Institute of Human Genetics, Bioinformatics Division, Carver College of Medicine, University of Iowa, Iowa City, United States of America; 3 Quantitative Biology Centre, Eberhard Karls University of Tübingen, Tübingen, Baden-Württemberg, Germany; 4 Department of Biomedical and Health Informatics, The Children’s Hospital of Philadelphia, Perelman School of Medicine, University of Pennsylvania, Philadelphia, Pennsylvania, United States of America; 5 Research Informatics Core, University of Illinois at Chicago, Chicago, Illinois, United States of America; 6 Integrative Bioinformatics Support Group, National Institute of Environmental Health Sciences, Durham, North Carolina, United States of America; 7 Bioinformatics Core, Purdue University, West Lafayette, Indiana, United States of America; 8 Department of Computational Sciences, The Jackson Laboratory for Genomic Medicine, Farmington, Connecticut, United States of America; Whitehead Institute for Biomedical Research, UNITED STATES

## Abstract

Life scientists are increasingly turning to high-throughput sequencing technologies in their research programs, owing to the enormous potential of these methods. In a parallel manner, the number of core facilities that provide bioinformatics support are also increasing. Notably, the generation of complex large datasets has necessitated the development of bioinformatics support core facilities that aid laboratory scientists with cost-effective and efficient data management, analysis, and interpretation. In this article, we address the challenges—related to communication, good laboratory practice, and data handling—that may be encountered in core support facilities when providing bioinformatics support, drawing on our own experiences working as support bioinformaticians on multidisciplinary research projects. Most importantly, the article proposes a list of guidelines that outline how these challenges can be preemptively avoided and effectively managed to increase the value of outputs to the end user, covering the entire research project lifecycle, including experimental design, data analysis, and management (*i*.*e*., sharing and storage). In addition, we highlight the importance of clear and transparent communication, comprehensive preparation, appropriate handling of samples and data using monitoring systems, and the employment of appropriate tools and standard operating procedures to provide effective bioinformatics support.

## Author summary

The article we wrote draws from our experience in core support facilities and highlights 10 best practices that individuals who apply information technology approaches to biological, medical, and health research should consider when providing support to individuals who generate data for this research in the lab. As interdisciplinary approaches are increasingly being utilized within the biological and medical sciences, effective collaboration and support between the aforementioned parties is crucial to promote the quality and integrity of research. These practices highlight the importance of quality control, comprehensive reporting, effective communication, and more in the production of quality data as well as the promotion of effective collaboration.

## Introduction

Because of the technological boom, life scientists are increasingly turning to high-throughput sequencing in their research programs and generating enormous volumes of data [1]. These projects are characterized by the use of specialized computational and tools to analyze the generated data, highlighting the need for interdisciplinary services and/or deep collaborations between primary data-generating researchers and bioinformaticians [1]. This trend has resulted in the establishment of both commercial and departmental (core) bioinformatics support facilities worldwide [2]. Because these facilities provide support to data-generating researchers in their data analysis and reporting, bioinformaticians in these facilities may inevitably encounter erroneous datasets (i.e., low-quality datasets primarily caused by experimental failures such as inadequate experimental design, improper sample collection and processing, sample contamination, degradation, sequencing, hybridization, library preparation, equipment and reagent failures, and more). When faced with erroneous data, bioinformaticians may be left without the necessary resources to address the associated challenges (*e*.*g*., which analysis method to employ). In essence, this highlights the importance of effective collaboration between bioinformaticians and data-generating researchers to provide effective support and analysis [3].

In this addition to the “Ten Simple Rules” series, we propose 10 rules to facilitate bioinformaticians in providing effective research support. These rules were developed based on extensive experiences of bioinformaticians working in core facilities and ordered to reflect the natural sequence of events in a project’s lifetime (project development, data collection and generation, and data analysis).

These rules can be scaled to both small single-site and large collaborative research projects and are therefore discussed as such. With the understanding that core facilities receive research projects at different stages of the project lifecycle, not all rules can always be implemented; however, these rules represent best practices that should be followed as much as possible to ensure the quality and integrity of all data collected and generated within a given research project. By implementing the following rules, bioinformaticians who routinely provide bioinformatics support to data-generating researchers can work to establish more realistic expectations for analysis while improving the quality and value of outcomes, owing to improved communication, experimental design, record keeping, data management and analysis. In addition, these rules discuss how to prevent the production of erroneous data as well as how such data can be treated.

## Project development

### Rule 1: Collaboratively design experiment

Successful bioinformatics analyses are dependent on appropriate experimental design, as previously described [4]. A good experimental design starts with a well-defined hypothesis and covers sample strategies (e.g., number and frequency), data handling, and data reporting. The experimental design should aim to reduce the types and sources of variability, increase the generalizability of the experiment, and make it replicable and reusable [4]. It is both easier and more cost efficient to identify and correct experimental design issues ahead of time than to address deficiencies thereafter. Thus, discussion between data-generating researchers and bioinformaticians is highly desirable and should occur as early as possible during project development and experimental design. Even so, bioinformaticians may not always have the luxury to provide input on experimental design. In such cases, it may be beneficial to request the experimental design and highlight concerns that may be of significance during data analysis.

During the experimental design discussions, a number of issues should be addressed, including cost, confounding batch effects, effect size, technical and biological replicates, sample integrity and purity, and controls. Researchers may be tempted to conduct many comparisons within the framework of one experiment containing large sample sizes (typically by sacrificing biological replicates). Therefore, it is crucial to discuss the critical role of appropriate sample sizes and replicates (biological and technical) [5], gain an understanding of the variables being investigated, and discuss the importance of avoiding confounding batch effects. If multiple samples or conditions are included in a project, batches should be constructed in a manner that evenly or randomly distributes experimental conditions across all the batches and processes during each experimental stage [6]. Similarly, the expected effect size of the test conditions should be carefully considered, as researchers may make unspoken assumptions about model system alterations while failing to plan for adequate replication to measure small effects [7].

### Rule 2: Manage scope and expectations

Successfully executed experiments are associated with attentive experimental design and clear communication [8]. Like Rule 1, communications regarding the potential limitations and pitfalls of a project (including technology, resources, and analysis) should occur prior to conducting the experiments. These communications should strive to eliminate extraneous technical detail without oversimplifying the topics (providing appropriate reference materials where required) [8]. Topics that should be covered include the employed wet and dry laboratory workflows (transparency should be provided from both sides) and, to avoid dissatisfaction, the expected and realistic turnaround times (it may be beneficial to clarify that these estimates refer to the time following receipt of data). In these initial communications, it is crucial to clarify the methods and responsible persons of future communications. To ensure that communications are clear and effective, a written analytical study plan (ASP) outlining the aforementioned topics should be prepared and agreed upon by all involved parties. Employed workflows should be documented and appropriately shared to enable bidirectional knowledge transfer for future reference. The ASP should be comprehensive and refer to the experimental design. It should also include the agreed upon timelines, the exact deliverables, and an alternative plan, in case the original data analysis plan is deemed insufficient.

To provide effective support and deliver the scientific vision of a project, scope management is critical [9]. The primary scope management patterns to monitor are (1) “scope grope,” in which a project takes an undefined path with no sight of completion, resulting in wasted resources without impact; (2) “scope swell,” in which the project expands rapidly without thoughtful allocation of resources and time, resulting in stress on the core and affecting the number of other projects which can be supported; and (3) “scope creep,” in which a project expands slowly but significantly, resulting in delayed project delivery, loss of impact, and over-consumption of planned resources. To flexibly manage the scope of a project and the expected outcome, universal adoption of the project management methodologies (including organizing resources, setting key milestones, and communicating to-go/not-to-go plans) is crucial and one of the primary aims of the developed ASPs. Ultimately, all involved parties should understand the proposed research vision and associated methodologies. The ASP serves to promote (1) easy sharing and storing of the study information and experimental design and (2) easy tracking of the project from wet to dry laboratory. Clear communication is thus imperative to providing effective support because it enables mutual knowledge transfer and understanding.

### Rule 3: Define and ensure data management

When receiving new support projects, it is critical to define the scope of data management required and set measures to ensure this management. A comprehensive data management plan (DMP) can be used to achieve this in projects involving high-throughput technology and data generation. Typically, a core facility should have a general standard operating procedure for data management that covers data handling during active analysis followed by long-term storage and backup. Because a core support facility typically deals with data owned by another party, a core's responsibility may only extend to secure storage of data and results for the client (short and long term), whereas final data sharing is the data owner’s responsibility. In many cases, however, data-generating scientists may call upon bioinformaticians to facilitate the development of a DMP for a research endeavor. As bioinformaticians in core facilities, it is crucial to communicate the importance of comprehensive DMPs to data owners. Similarly, bioinformaticians should be aware and communicate the extent of their core’s DMP.

Guidelines to developing a good DMP have been previously described [10]. Aspects to consider when developing a DMP include determining the legal, ethical, and funder’s requirements associated with the data; identifying the types of data to be collected; identifying the standards and ontologies that will be employed; and determining how data will be organized, quality controlled, documented, stored, and disseminated [10]. In addition, core facilities should also consider defining applicable data handling policies and preparing data management budgets.

Comprehensive DMPs aim to address the ethical, governance, and resource requirements associated with the data; promote findable, accessible, interoperable, and reusable (FAIR) research [11]; and consider associated data security, access, and the backup concerned. Ultimately, the DMP provides assurance for the long-term preservation and accessibility of the generated data [12]. Like the experimental designs, DMPs can be collaboratively developed or selected by data-generating researchers and bioinformaticians. However, bioinformatics core facilities may also choose to develop a standard DMP that can be adjusted as required for individual projects.

## Data collection and generation

### Rule 4: Manage the traceability of data

Traceability of all samples and data in a research project is a crucial component of effective bioinformatics support [13]. Traceability should be comprehensive and encompass sample acquisition and processing, as well as data generation, analysis, storage, and reporting [13]. The best-case scenario is a database management system that is maintained by and accessible to both the data-generating scientists and bioinformaticians. In practice, this kind of system is called a laboratory information management system (LIMS) and may be implemented to simplify the traceability of samples and data, thereby reducing human error and the production of erroneous data [14]. If a LIMS is not feasible, a shared cloud-based resource may serve the same purpose [15].

These systems should enable the production of reliable results at a faster rate than manual systems and enable data tracking from sequencing runs over time and across experiments in order to improve efficiency and trace down potential errors [14]. Additionally, these systems also promote quality control by highlighting failed samples and identifying the accountable parties. Enabling sample and data traceability is ultimately one of the most efficient ways to identify sources and prevent production of erroneous data [14]. Notably, bioinformaticians may not always be part of a sequencing core and are therefore dependent on data owners providing accurate information. Comprehensive DMPs (see **Rule 3**) may need to account for the precise setup applicable to individual clients.

### Rule 5: Determine how and what metadata are reported

In order for bioinformaticians to conduct appropriate downstream data analysis of an experiment, the associated metadata must be provided. Metadata should be as complete as possible and should include the experimental variables of interest, all aspects of sample handling, known or suspected sources of batch variables, and laboratory mistakes such as sample mislabels and swaps [16]. To account for the aforementioned considerations, an effective system should be implemented to ensure comprehensive metadata reporting. Ideally, data-generating facilities should adopt a system to enable tracking of critical information related to the experiments and pass this information to the bioinformatics core (see Rule 4). Because many research groups may not have the luxury of an LIMS, the data-generating researchers and bioinformaticians should propose or develop standardized worksheets or web-based submission forms for metadata reporting, which designate required and optional fields [16]. In the absence of a standardized approach, metadata reporting may be provided in various forms (e.g., spreadsheets, handwritten notes, etc.); however, these often lack critical batch information and other insights from the wet laboratory experimental practices. If possible, it might be worth planning from the onset where data will be made publicly available. In many cases, the data will be deposited in an existing public repository; therefore, knowing the structure and depth of metadata collection required for the repository is crucial. For smaller-scale studies, metadata templates provided by the repository can be used to record samples so that everything is already prepared for final submission as well.

In the interest of producing interoperable research, metadata reporting should adhere to experiment-specific reporting guidelines, such as Minimum Information About a Microarray Experiment (MIAME) [17], Minimum Information required for a DMET Experiment (MIDE) [18], Minimum Information About a Proteomics Experiment (MIAPE) [19], and more. These can be accessed through FAIRsharing (https://fairsharing.org/) (a standards-housing resource), BioSchemas (https://bioschemas.org/), and the Global Alliance for Global Health (GA4GH) (https://www.ga4gh.org/). Similarly, metadata ontologies can be found in online repositories such as Bioportal (https://bioportal.bioontology.org/) and Ontology Lookup Service (https://www.ebi.ac.uk/ols/index). Selecting and employing appropriate reporting standards should be covered in the DMP (**Rule 3**) and may be required by journals or funders. Reporting standards ensure that researchers adhere to internationally set standards during their experimental procedures. Moreover, employing data reporting standards, helps to promote reuse and comparison to previously conducted studies [20]. Ultimately, this ensures that both researchers and their community reap the maximum benefit from their collected and generated data.

### Rule 6: Coordinate data and internet security

Providing assurances that data are both secure and stable is an important aspect of providing effective bioinformatics support [21]. Although these aspects are typically addressed by an information technology (IT) department or system administrator, it is crucial to communicate the particular requirements with the responsible person(s) and may be an important consideration in resource- or capacity-limited facilities.

Data security refers to the prevention of harmful cyber-attacks and unoptimized internet security issues, as well as the setting of data access and transfer limitations [21]. Generally, the individuals with access to research data should be limited to parties with relevant responsibility and accountability. Cases pertaining to personal data, particularly patient data, may require auditing of data access as well. The aspects that need to be considered when safeguarding data to maintain quality, include (1) confidentiality (maintaining access and transfer); (2) integrity (ensuring information is accurate, valid, and reliable); (3) availability (resources and support are available); (4) accountability (actions can be attributed to relevant parties); and (5) provenance (origin and history of data are known and well defined).

Internet security refers to the use and stability of the internet, which is employed to manage and analyze data associated with high-throughput experiments [21]. To address the computational challenges (e.g., central processing units [CPUs], memory, storage) associated with high-throughput data analysis, cloud computing has emerged as the leading solution. In these cases, the importance of ensuring data and internet security are further emphasized. As a result, cloud users have to rely heavily upon the service providers for data privacy and security protection; therefore, data backups and recovery plans should be maintained and monitored.

GA4GH has released a data security toolkit (www.ga4gh.org/genomic-data-toolkit/data-security-toolkit/) for genomics and health-related data sharing. This toolkit consists of recommendations for privacy and security safeguards and procedures for maintaining proper access and fidelity of data. Useful tips to support this maintenance include (1) developing access control documents (that are reviewed and updated periodically); (2) implementing data verification and reporting processes; (3) implementing risk management strategies; (4) establishing strong working relationships with local IT support; (5) implementing regular maintenance and upgrade processes; and (6) implementing real-time server monitoring systems and maintaining security certificates associated with maintained sites and software [21].

## Data analysis

### Rule 7: Control data quality throughout the project lifecycle

Quality control is inarguably the most important component of high-throughput experiments. This pertains to both the quality control of data generated by high-throughput technologies to enable downstream analysis as well as the quality control of the generated results to make reliable scientific inferences. Quality control occurs throughout the project lifestyle (i.e., during each implemented standard operating procedure of a workflow). In some cases, experimental failures may be inevitable; therefore, data quality control needs to be performed by the bioinformatics core at various stages of processing. In commercial facilities, quality control is typically ensured by a quality control manager, who maintains the quality control processes, conducts root cause analyses, and implements corrective and preventive actions [22,23]. In core facilities, quality control is the responsibility of all bioinformaticians.

Documenting the implemented quality control procedures is a crucial component of this rule [3]. When reviewing data quality, it is essential for a bioinformatician to be able to refer to the quality control procedures implemented to appropriately interpret the metrics and, subsequently, conduct suitable analysis. Importantly, quality control needs to be implemented at both sample level and cohort level. Whereas the former identifies inadequate sample data, the latter identifies outliers in the overall data of the cohort. Following the interpretation, bioinformaticians should effectively communicate quality metrics to primary investigators, to identify potential issues, make go or no-go decisions, and design the proper analytical approaches for addressing their research objectives. The selection of appropriate quality control processes, gates, and values play an important part in the downstream analysis of high-throughput omics data [24]. Where necessary, it may be useful to follow developers’ recommendations. Such processes eliminate or reduce erroneous data within a data set and may be adjusted to salvage as much data as possible. Balancing the data quality parameters and statistical power is key, thus, one should proceed with caution. With this in mind, the implementation of Rules 1 and 2 is crucial to appropriately handle these cases, especially in high-throughput research, as the selection of quality control processes and parameters needs to be appropriately justified in research communications. The incorporation of the previous rules aims to facilitate quality control, which highlights the importance of implementing this rule to maintain research and experimental integrity.

### Rule 8: Identify suitable computational tools for data analysis

An important component of providing effective bioinformatics support is conducting research that is reproducible and reusable. When conducting data analysis, it is crucial to employ appropriate bioinformatics methods (tools and resources) and statistical models that deliver reliable inferences from the data. As is the nature of the science, several bioinformatics tools have been developed and proposed for application in high-throughput experiments. However, no tool is expected to be the best for all situations, though tools can be recommended for repeated or common workflows. So how do we determine an appropriate tool to use? Firstly, we have to size up the data characteristics with the aims of the analysis; attention needs to be given to the strengths and limitations of a given tool for the analysis at hand. Analysis using tools that are of academic standard are usually a good place to start; however, we can also look to which tools are employed by similar projects. In addition, several other features may be investigated to identify appropriate tools; these include whether the tool is supported by the developers, whether the tool gains active support in relevant question and answer (Q&A) forums, whether the tool is open source, documented, and version controlled, and, depending on the bioinformaticians’ experience, whether the tool is easily installable, executable, and parallelizable. It may be useful for a core facility to have a procedure or criteria in place for the use of new tools when analyzing high-throughput data.

Notably, when implementing a selected method, significant attention needs to be given to the measurement of *p*-values and estimating false discovery rates (FDRs) due to the violation of assumptions of statistical models and dependency among the hypotheses tested [25, 26]. Ultimately, the use of specific tools, statistical models, and values adds an important layer of understanding to the overall research project. This enables and promotes future collaborations, allows others to critically evaluate the research at hand, and increases the credibility of the findings and allows the researchers themselves to identify the limitations and strengths of their research and generated data.

### Rule 9: Track, record, and confirm workflow changes

Establishing methods to track and record changes to workflows can go a long way in improving bioinformatics support services and ensuring quality control during data analysis. In practice, this is called verifying and validating workflow changes and is typically required to adhere to international quality management standards, such as those proposed by the International Organization for Standardization (www.iso.org/home.html).

Because of technical or software updates, adjusted project requirements, or process improvements, workflows may be altered from time to time. Whenever such alterations occur or new workflows for specific analyses are developed, it is important to independently verify and validate them. Technical verification and validation are not only necessary to ensure that new or altered workflows are working as expected and are fit for purpose but also to ensure that the workflow can be maintained while handling data inputs of different sizes and types and adapting to different technical landscapes [27]. Validating workflow alterations and communicating these alterations to collaborators or clients are essential for reproducible research and scope management, as described in Rule 2.

Deviations refer to any observed events in data analysis procedures that are exceptions or alterations from specifications or acceptance criteria [28]. These may include out of specification, tolerance or trend results, deviations from an approved standard operating procedure, test method, validation protocol or ASP, and software failures [28]. Maintaining a system by which these deviations can be reported and monitored functions as an important component of both metadata reporting and quality control and maintenance [23]. These reports supplement root cause analysis and corrective and preventive action in commercial facilities, as described in **Rule 8**. These practices may also prevent consistent production of erroneous data and simplify error tracking. A bug tracking and change management system would be critical in core facilities in which multiple people may be working on complex workflows/pipelines at the same time. Ultimately, such practices also provide assurances in the core facilities’ practices and capacity to collaborators and clients.

### Rule 10: Repurpose the data

In cases wherein erroneous data are produced, researchers may choose to terminate a project to save research funds or conform to service agreements. However, the data produced may yet be informative. Before terminating a project, there should be clear communication (as outlined in Rule 2) between the bioinformaticians and primary researchers; the cost of the experiments may be weighed up against the outputs that may still be desirable and relevant to the end user, highlighting the importance of effectively communicating the pros and cons of the decision. A detailed sample, design, and tool review may inform the aforementioned decision. A sample review includes the review of LIMS data, the cohort, the batches, the adaptors, and identifiers. This review aims to identify where experimental failures occurred or where erroneous data were produced. On the other hand, a design review includes cohort composition analysis, power analysis, and batch identification and confounding. This review aims to identify faults within the experimental design; these may be adjusted or tightly regulated in the future. Lastly, in a computational tool review, tools are verified and validated using test data, and maintenance and suitable support for the tools are identified.

Importantly, with regards to low-quality data or marginal data (datasets closer to the lower limit of qualification and acceptability, i.e., datasets that barely exceed the minimum requirements for downstream analysis), there are 2 ways in which projects can be continued: (1) using partial data or (2) repurposing the data. The former refers to the use of replicates with enough depth and quality to answer the initially posed research questions, albeit at a smaller scale. In case of the latter, although the data generated may not be sufficient for answering the initial research question, it may be appropriate to repurpose the data by answering an additional or alternative research question within the scope of the project. Importantly, marginal data can also be used for improvement of workflows, procedures, and overall quality of similar studies in the future and could be used to guide future experimental procedures and designs.

## Conclusion

High-throughput data play a key role in expediting scientific discoveries and rapidly providing scientific understanding to improve human health [1]. Therefore, effective collaborations with bioinformatics cores are essential to modern bioscientific research. Theoretically, data-generating and bioinformatics cores may be seen as 2 separate entities, functioning separately in the same research project; however, they are intrinsically connected and highly dependent on each other to function effectively. Effective bioinformatics collaborations aim to conduct quality research and reduce the production of marginal data. To meet these aims, these collaborations require clear communications between the 2 entities of the collaboration; appropriate reporting and documentation that can be referred to in the future; the appropriate collection and reporting of data and metadata; appropriate quality control, validation, verification, and deviation reporting procedures; and the use of appropriate technology and computational tools that are specific to both the data generated and the research questions being investigated. Although majority of the rules apply to maintaining and ensuring data quality, taking the same approach to data exploration and analysis stages can result in analyses that are inflexible and might miss important but unexpected findings. Having an analysis structure that is resistant to change can tend to prefer a stock analysis rather than adapting to the early stage findings. Building in consultation checkpoints between bioinformaticians and data-generating scientists through all stages of the project lifecycle is crucial to ensure that the best results are obtained. Again, the best rule to adopt and implement will depend on the nature of the study, but pointing out that some parts of the analysis are easier to predefine than others might be a useful addition.

Overall, effectively implementing any of these rules in bioinformatics support facilities will facilitate increased productivity, credibility, and satisfaction while simultaneously reducing erroneous data production and promoting high-quality research. Ultimately, the proposed rules ensure that information is reported and communicated correctly, at the highest quality, making it broadly beneficial.
